# Effect of therapeutic plasma exchange on tissue factor and tissue factor pathway inhibitor in septic shock

**DOI:** 10.1186/s13054-024-05142-4

**Published:** 2024-10-30

**Authors:** Klaus Stahl, Georg F. Lehner, Pedro David Wendel-Garcia, Benjamin Seeliger, Thorben Pape, Bernhard M. W. Schmidt, Heiko Schenk, Julius Schmitt, Andrea Sauer, Lennart Wild, Konrad Peukert, Christian Putensen, Christian Bode, Michael Joannidis, Sascha David

**Affiliations:** 1 Department of Gastroenterology, Hepatology, Infectious Diseases and Endocrinology, Hannover Medical School, Hannover, Germany; 2 Division of Intensive Care and Emergency Medicine, Medical University Innsbruck, Innsbruck, Austria; 3 Institute of Intensive Care Medicine, University Hospital Zurich, Zurich, Switzerland; 4 Department of Respiratory Medicine and German Centre of Lung Research (DZL), Hannover Medical School, Hannover, Germany; 5 Department of Nephrology and Hypertension, Hannover Medical School, Hannover, Germany; 6 Department of Anaesthesiology and Intensive Care Medicine, University Hospital Bonn, Bonn, Germany

**Keywords:** Extracorporeal treatment, Sepsis, Plasmapheresis, Tissue factor, Tissue factor pathway inhibitor, Coagulation, Apheresis, Precision medicine

## Abstract

**Background:**

Coagulopathy is part of the pathological host response to infection in sepsis. Higher plasma concentrations of both tissue factor (TF) and tissue factor pathway inhibitor (TFPI) are associated with occurrence of disseminated intravascular coagulation (DIC), multi-organ dysfunction and increased mortality in patients with sepsis. Currently no treatment approaches specifically targeting this axis are available. We hypothesize that therapeutic plasma exchange (TPE) might limit this coagulopathy by restoring the balance of plasma proteins.

**Methods:**

This was a pooled post-hoc biobank analysis including 51 patients with early (shock onset < 24 h) and severe (norepinephrine dose > 0.4 μg/kg/min) septic shock, who were either receiving standard of care treatment (SOC, n = 14) or SOC + one single TPE (n = 37). Plasma concentrations of TF and TFPI were measured both at- and 6 h after study inclusion. The effect of TPE on concentrations of TF and TFPI was investigated and compared to SOC patients. Further, baseline TF and TFPI concentrations were used to modulate and predict clinical response to adjunctive TPE, indicated by longitudinal reduction of lactate concentrations over the first 24 h following study inclusion.

**Results:**

TPE led to a significant reduction in circulating concentrations of both TF and TFPI while no difference was observed in the SOC group. Relative change of TF within 6 h was + 14 (-0.8 to + 30.4) % (p = 0.089) in the SOC and −18.3 (−32.6 to −2.2) % (p < 0.001) in the TPE group (between group p < 0.001). Similarly, relative change of TFPI was + 14.4 (−2.3 to + 30.9) % (p = 0.076) in the SOC and −20 (−32.8 to −7.9) % (p < 0.001) in the TPE group (between group p = 0.022). The ratio of TF to TFPI remained unchanged in both SOC and TPE groups. SOC patients exhibited an increase in lactate over the initial 24 h when TF and TFPI concentrations were higher at baseline. In contrast, patients undergoing TPE experienced a sustained longitudinal reduction of lactate concentrations across all levels of baseline TF and TFPI elevations. In a multivariate mixed−effects model, higher baseline TF (p = 0.003) and TFPI (p = 0.053) levels led to greater longitudinal lactate concentration reduction effects in the TPE group.

**Conclusions:**

Adjunctive TPE in septic shock is associated with a significant removal of both TF and TFPI, which may contribute to the early hemodynamic improvement observed in septic shock patients receiving TPE. Higher baseline TF (and TFPI) plasma concentrations were identified as a putative predictor of treatment response that could be useful for predictive enrichment strategies in future clinical trials.

**Supplementary Information:**

The online version contains supplementary material available at 10.1186/s13054-024-05142-4.

## Background

Sepsis is a life-threatening organ dysfunction caused by a dysregulated host response to infection [1]. This overwhelming host response, characterized by cytokine release, attraction of inflammatory cells, global endothelial activation and permeability as well as coagulopathy, is the key driver of morbidity and mortality [2]. While recognizing that early ICU admission, adherence to the 1 h bundle resuscitation measures, strict source control and anti-infective efforts certainly have improved survival in septic shock patients [3], mortality associated with refractory septic shock nevertheless remains high in the absence of a specific intervention targeting the pathological host response, [4]. Numerous therapeutic interventions specifically targeting this host response have been tested in trials but all have failed so far [5]. The vast majority of them as focused on the immune response but not on alterations of endothelial or coagulatory function. Sepsis-induced coagulation (SIC) and disseminated intravascular coagulation (DIC) are complex pathophysiological processes that might yield further potential for interventional trials. Sepsis triggers a marked release of Tissue factor (TF), a potent initiator of the coagulation cascade, and high levels of this pro-coagulatory factor propagate SIC and DIC, microvascular- and consecutive multi-organ dysfunction [6–9]. In contrast, Tissue factor pathway inhibitor (TFPI) is primarily recognized as an anti-coagulatory factor. It inhibits TF activity, thereby reducing generation of thrombin and downstream fibrin formation [10]. Initial animal experiments yielded promising findings where administration of TFPI improved survival in non-human primates with septic shock [10]. However, a subsequent phase-3 study using recombinant TFPI (i.e. Tifacogin) yielded disappointing results. It showed no effect on mortality and instead observed an increased rate of bleeding in patients with severe sepsis and elevated international normalized ratio (INR) [11]. Of note, in a recent analysis from septic shock patients, high TFPI levels were associated with overall sepsis disease severity and particularly with acute kidney injury (AKI), liver dysfunction as well as high levels of D-Dimer and lactate [12]. Moreover, non-survivors had a particularly low TF/TFPI ratio due to higher TFPI levels compared to survivors [12], suggesting altogether a potentially harmful effect of high TFPI concentrations in septic shock.

Our group has recently described the effect of TPE against healthy fresh frozen plasma (FFP) as an additive treatment strategy in early (onset < 24 h) and severe (norepinephrine (NE) dose > 0.4 μg/kg/min) septic shock [13–15]. In both, an uncontrolled (EXCHANGE-PILOT study) [13] and randomized controlled (EXCHANGE-1 study) [14, 15] design, we observed rapid stabilization of hemodynamics, indicated by reduction of vasopressor requirement and blood lactate concentrations. We believe that these positive surrogate effects might be attributed to both removal of harmful circulating molecules (pro-inflammatory cytokines, permeability and pro-coagulant factors) [13, 15–17] as well as simultaneous replacement of protective plasma proteins (anti-inflammatory cytokines, immunoglobulins, anti-permeability and anticoagulatory factors) [13, 15–18], that are consumed by the disease process [19]. While recent meta-analyses have suggested that TPE may improve survival in septic shock [20–22], it remains unclear which subset of patients might benefit most from this intervention.

In this study, we investigated the effect of TPE on concentrations of both TF and TFPI. Further, baseline TF and TFPI concentrations were used to predict clinical response to TPE, indicated by early reduction of lactate concentrations. We hypothesized that TPE might modulate concentrations of both TF and TFPI and that the profile of these two disease mediators might identify septic shock patients potentially benefiting most from adjunctive TPE.

## Methods

### Study population

**Fig. 1:**
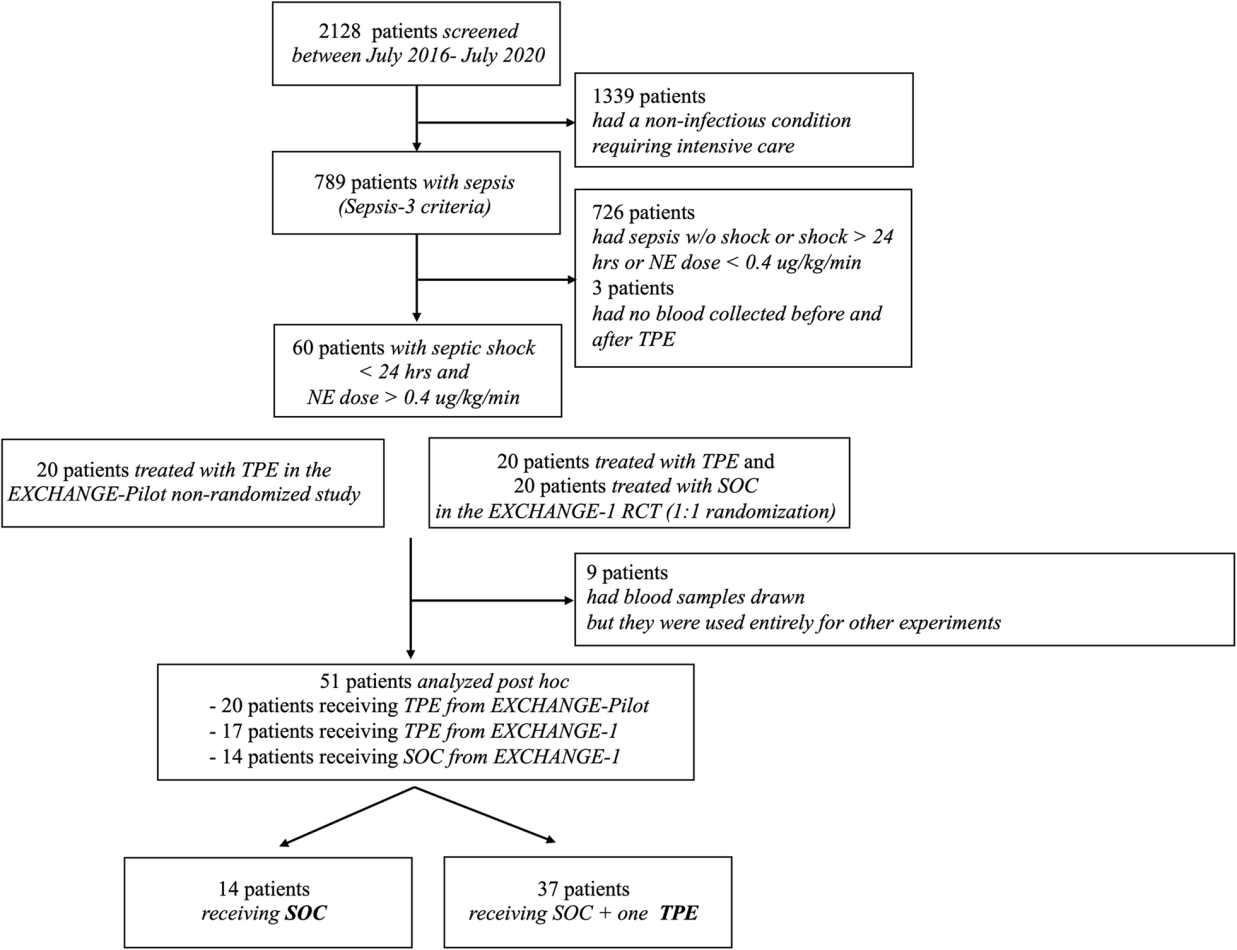
Flow chart of study participants. Shown are screening, inclusion and analysis of patients. Inclusion criteria were early (< 24 h) and severe (norepinephrine (NE) dose ≥ 0.4 μg/kg/min despite adequate fluid resuscitation) septic shock. The study compared standard of care (SOC) with SOC + a single therapeutic plasma exchange (TPE). This was a post-hoc biobank analysis pooled from both a pilot non-randomized prospective observational study (EXCHANGE-Pilot) and an open-label RCT (EXCHANGE-I)

This was a post-hoc biobank analysis, where data and bio-samples were acquired from two consecutively performed studies, a prospective single center non-randomized observational study (EXCHANGE-Pilot) [13] and an open label RCT (Exchange-1), at two university hospitals in Germany [14, 15]. In both studies together, we screened 2128 patients from July 2016 to July 2020 for the presence of sepsis per SEPSIS-3 definition [1] (Fig. 1). A total of 60 patients with early (onset < 24 h) and severe (requiring a NE dose of ≥ 0.4 μg/kg/min) septic shock was recruited in total in the EXCHANGE-Pilot (n = 20) and Exchange-1 (n = 20 vs. 20) study. In the EXCHANGE-Pilot study, due to its non-randomized nature, all 20 patients received TPE treatment. In contrast, in the EXCHANGE-1 study, 20 patients were assigned to the intervention group (SOC in addition to one single session of TPE) and 20 patients to the control group (SOC only). Out of 60 original participants, only 51 participants, of whom 14 received SOC and 37 received one additional TPE, were analyzed in this present post-hoc biobank analysis because blood samples had been used for other experiments in the remaining patients. All patients were treated according to the 2016 Surviving Sepsis Campaign (SSC) guidelines [23]. The protocol including inclusion and exclusion criteria as well as endpoints and statistical analysis has recently been published [14, 15]. The studies were conducted according to the principles of the Declaration of Helsinki and was approved by the Institutional Review Board of the Hannover Medical School (No. 2786–2015 and No. 8852_MPG_23b_2020) and the University Hospital Bonn (No. 024/20). Written informed consent was provided by participants or their legal representatives prior to enrollment and randomization, respectively. Demographic and clinical data were obtained immediately at study-inclusion (e.g. before TPE in the TPE group).

### Inclusion & exclusion criteria

Eligible patients were adults admitted to the ICU with early septic shock (onset of vasopressor use < 24 h prior to screening), and a NE requirement of ≥ 0.4 µg/kg/min despite adequate intravenous fluid resuscitation (≥ 30 ml/kg bodyweight crystalloids). Exclusion criteria included age < 18 years, pregnancy or breast feeding, end-stage chronic kidney disease, and presence of a directive to withhold life-sustaining treatment.

### Randomization and intervention

Randomization was concealed via sealed opaque envelopes. Patients were assigned in a 1:1 ratio to SOC or SOC in addition to one single session of TPE against FFP. TPE had to be performed within 6 h following randomization. Vascular access was established by venous insertion of an 11-French two-lumen hemodialysis catheter. We performed only one singular TPE session, as hemodynamic improvements were achieved only with the first TPE in a previous pilot study [24]. Centrifugal TPE was performed against a fixed dose of 12 units of FFP, therefore total volumes exchanged varied between 1 and 1.5 times the plasma volume. Mean duration of TPE sessions was 121 ± 37 min. Anticoagulation during TPE was performed by regional citrate infusion. Post filter calcium concentration was checked 15 min after starting treatment as regulated by a local protocol at our institution. Citrate flow rate was adjusted to target post filter ionized calcium concentrations of 0.5 to 0.6 mmol/l. Additionally, systemic blood gas analysis was performed every two to four hours to exclude electrolyte and acid base dysbalances. Immediately before TPE treatment implementation, anti-allergic intravenous prophylaxis with ranitidine and clemastine was administered to all patients. In patients with acute kidney injury (AKI) requiring renal replacement therapy, hemodialysis was interrupted for the duration of TPE. NE dose was titrated every 10–15 min to maintain a mean arterial pressure (MAP) above 65 mmHg.

### Blood sample collection and measurement of parameters

Blood samples were drawn at study inclusion and 6 h following study inclusion. For blood sampling 10 ml monovettes containing 1 ml of 0.13 mol/l sodium citrate were used and plasma was obtained by centrifugation, divided into aliquots and stored at –80° until assayed.

Levels of TF and TFPI were determined by using Human Tissue Factor SimpleStep ELISA® (ab220653, abcam) and Human TFPI SimpleStep ELISA® (ab274392, abcam), respectively. Plasma samples were appropriately diluted and assays were performed according to the manufacturer’s instructions. The measurements were performed in Tecan i-control infinite 200 (Tecan Austria, Gröding, Austria) at 450 nm with reference at 570 nm.

### Statistical analysis

Data were presented as median with interquartile range (IQR). Normality of data distribution was assessed prior with the D'Agostino-Pearson omnibus normality test and the Shapiro–Wilk normality test. Within-group effects between the chosen two time points (randomization, 6 h after randomization) were analyzed via paired-t-test or Wilcoxon signed-rank test as appropriate. Comparisons between groups were analyzed by means of Mann–Whitney U test. Patient characteristics at inclusion were compared by t-test, Fisher’s exact test or the Kruskal–Wallis test as appropriate.

Modelling of the effect of TPE on repeated-measures of lactate levels was approached by means of a linear mixed-effects model. Lactate measures were entered as outcome variable into the model, whereas TPE or SOC and time were entered as independent fixed effects including the interaction between both, finally per patient random intercepts were entered into the model. P values for individual fixed effects were obtained by Satterthwaite’s degrees of freedom method. In order to explore predictor variables for TPE effect, TF and TFPI were entered as additional fixed effects including a triple interaction term with TPE/ SOC and time, as well as all simple interaction terms between fixed effects. Model fit was assessed using a likelihood ratio test of the full model with the effects in question against a “null model”. Interaction terms were retained only if they were found to contribute to the model.

For all statistical analyses a two-tailed p-value < 0.05 was considered statistically significant. GraphPad Prism 7 (Graph Pad, La Jolla, CA, USA), SPSS Statistics Version 25 (SPSS Inc., Chicago, IL, USA) and the R environment for statistical computing version 4.1.2 (R Foundation for Statistical Computing, Vienna, Austria) were used for data analysis and graph generation.

## Results

### Cohort characterization

Demographics, disease severity, comorbidities, site of infection, and type of causative pathogen were comparable between SOV and TPE groups at study inclusion (Table 1**)**. Seventy-two percent of the patients were male, and the median (IQR) age was 54 (42–60) years. The most common site of infection were the lungs and abdomen, with the causative pathogen being identified in about 75% of cases. SOFA score at study inclusion was 17 [15–19] points. Shock severity was profound indicated by a norepinephrine (NE) dose of 0.660 (0.490–0.889) µg/kg/min and lactate concentration of 4.2 (2.7–7.3) mmol/L. All patients displayed signs of severe inflammation as indicated by high levels of C-reactive protein (CRP) and procalcitonin (PCT). Patients had an oxygenation index (P_aO2_ / F_IO2_) of 135 (95–223) mmHg, with 92% requiring mechanical ventilation. AKI with need for early renal replacement therapy (RRT) was present in 65% of the patients at time of inclusion.

**Table 1:** Demographic and clinical characteristics at study inclusion

Category	All n = 51	SOC n = 14	TPE n = 37	p
Age—years (median, [IQR])	54 [42–60]	57 [43–64]	53 [41–59]	0.562
Sex—n (%)				0.297
Female	14 (27.5)	2 (14.3)	12 (32.4)	
Male	37 (72.5)	12 (85.7)	25 (67.6)	
BMI—kg/m^2^ (median, [IQR])	25.7 [22.2–31.1]	25.5 [23.8–34.3]	25.9 [21.3–31.1]	0.239
Sepsis onset—n (%)				1
Ambulatory	31 (60.8)	9 (64.3)	22 (59.9)	
Hospital	21 (41.2)	5 (35.7)	15 (40.5)	
Side of infection—n (%)				
Pulmonary	32 (62.7)	9 (64.3)	23 (62.2)	1
Abdominal	14 (27.5)	4 (28.6)	10 (27)	1
Soft tissue	4 (7.8)	1 (7.1)	3 (8.1)	1
Endocarditis	2 (3.9)	0 (0)	2 (5.4)	1
Identified pathogen—n (%)				
Gram +	11 (21.6)	5 (35.7)	6 (16.2)	0.148
Gram-	15 (29.4)	3 (21.4)	12 (32.4)	0.513
Fungal	4 (7.8)	1 (7.1)	3 (8.1)	1
Viral	4 (7.8)	2 (14.3)	2 (5.4)	0.3
Mixed	4 (7.8)	0 (0)	4 (10.8)	0.565
Non-identified	13 (25.5)	3 (21.4)	10 (27)	1
SOFA score—points (median, [IQR])	17 [15–19]	18 [15–19]	17 [15–19]	0.969
Norepinephrine dose—µg/kg/min (median [IQR])	0.660 [0.490–0.889]	0.483 [0.443–0.624]	0.792 [0.559–1.056]	**0.033**
Lactate concentration—mmol/l (median, [IQR])	4.2 [2.7–7.3]	3.9 [2.9–5.5]	4.3 [2.7–9.5]	0.148
RRT—n (%)	33 (64.7)	9 (64.3)	24 (64.9)	1
Mechanical ventilation—n (%)	47 (92.2)	12 (85.7)	35 (94.6)	0.3
Oxygenation index (PaO_2_/FiO_2_)—mmHg (median [IQR])	135 [95–223]	167 [89–249]	128 [94–209]	0.7
CRP—mg/l (median [IQR])	286 [161–349]	313 [126–435]	267 [176–329]	0.312
PCT—µg/l (median [IQR])	27.6 [12.7–84.2]	32.7 [13.8–100.3]	23.4 [9.1–64.9]	0.84
WBC -10^3^/µl (median [IQR])	16.6 [6.5–24.9]	16.7 [5.1–18.2]	23.4 [9.1–64.9]	0.441

Given are demographic and clinical characteristics at the time of study inclusion for patients receiving standard of care treatment (SOC) as well as for patients receiving additive therapeutic plasma exchange (TPE). Values are presented as median (25% to 75% interquartile range) or if categorical as numbers and percentages

*BMI*, Body mass index: *CNS*, Central nervous system: *CRP*, C-reactive protein: *IQR*, Interquartile range: *PCT*, Procalcitonin: *RRT*, Renal replacement therapy: *SOFA*, Sequential Organ Failure Assessment: *WBC*, White blood cell

### Effect of TPE on tissue factor concentration

**Fig. 2:**
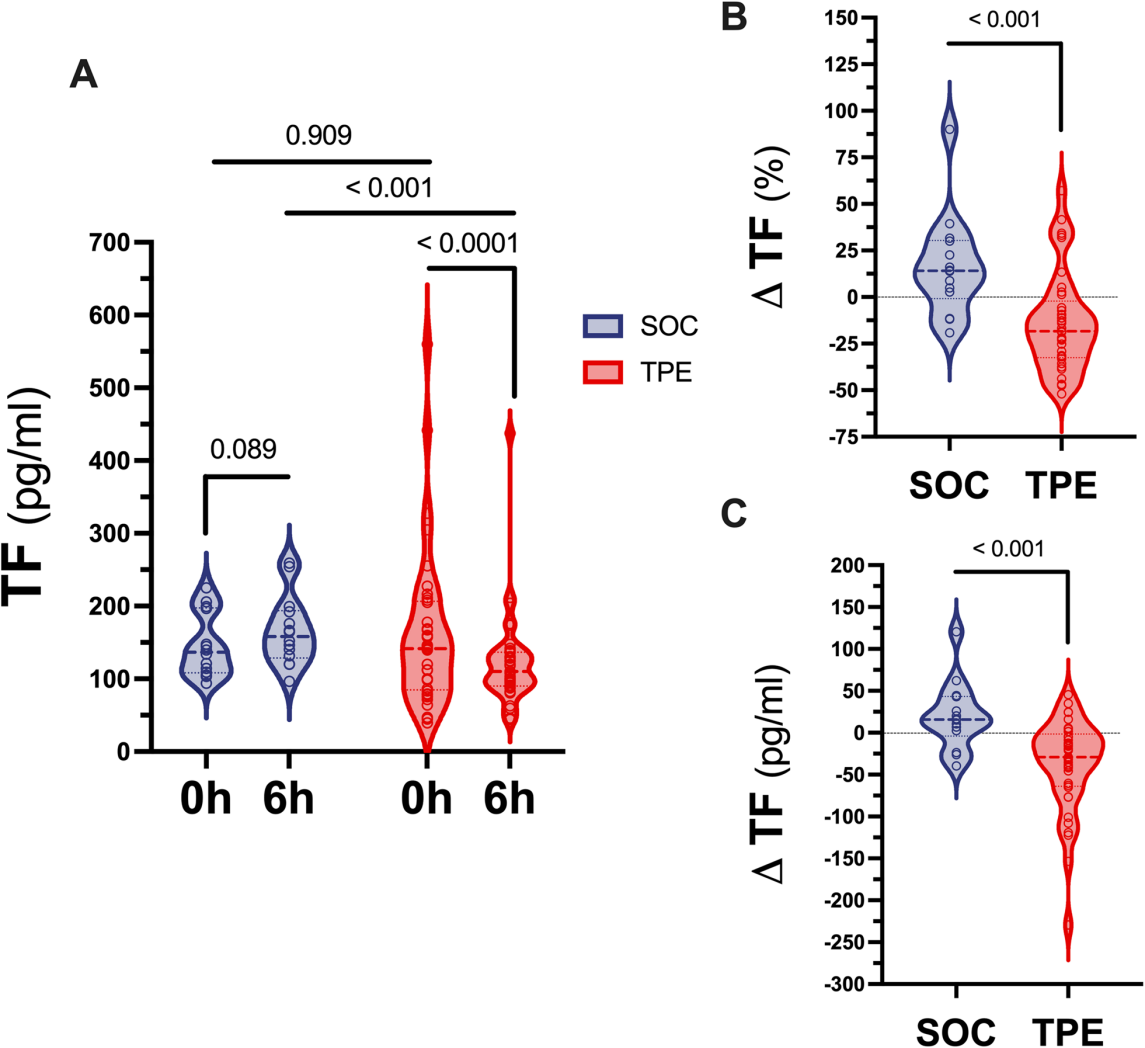
Effect of TPE on tissue factor concentrations. Violin plots showing tissue factor (TF) plasma concentrations at study inclusion and 6 h after study inclusion in patients with septic shock who received either standard of care (SOC) alone or SOC in combination with therapeutic plasma exchange (TPE) (**A**). Both relative (**B**) and absolute (**C**) change of TF concentrations over time are shown for SOC and TPE groups

Median (IQR) TF concentrations were comparably high in both SOC and TPE groups at baseline (SOC 0 h: 136.7 (108.2–197.4) pg/ml vs. TPE 0 h: 141.5 (84.8–206.6) pg/ml, p = 0.909, Fig. 2A). Six hours after study inclusion TF concentrations were lower in the TPE than in the SOC group (SOC 6 h: 158.3 (128.8–193.9) pg/ml vs. TPE 6 h: 110.3 (90.2–136.5) pg/ml, p < 0.001, Fig. 2A). While TF concentrations numerically even increased in the SOC group within 6 h (p = 0.089), they decreased in the TPE group (p < 0.001). Relative change of TF between 0 and 6 h was + 14 (−0.8 to + 30.4) % in the SOC and −18.3 (−32.6 to −2.2) % in the TPE group (p < 0.001) (Fig. 2B). This corresponded to an absolute change in TF concentrations of + 15.9 (−4 to + 43.3) pg/ml in the SOC and −29.2 (−64.1 to −1.8) pg/ml in the TPE group (p < 0.001) (Fig. 2C).

### Effect of TPE on tissue factor pathway inhibitor (TFPI)

**Fig. 3:**
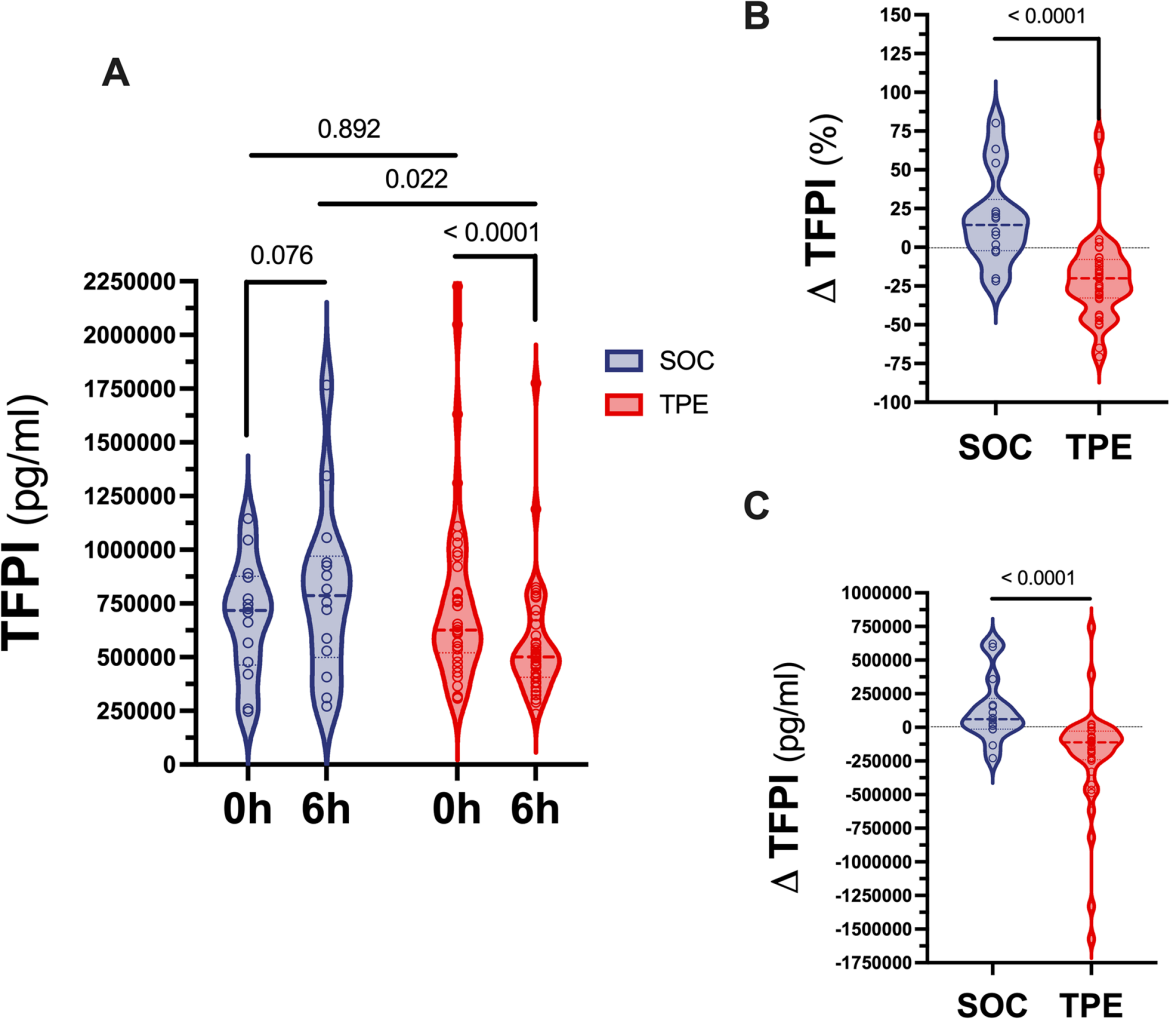
Effect of TPE on tissue factor pathway inhibitor concentrations. Violin plots showing tissue factor pathway inhibitor (TFPI) plasma concentrations at study inclusion and 6 h after study inclusion in patients with septic shock who received either standard of care (SOC) alone or SOC in combination with therapeutic plasma exchange (TPE) (**A**). Both relative (**B**) and absolute (**C**) change of TFPI concentrations over time are shown for SOC and TPE groups

TFPI concentrations were comparable in both SOC and TPE groups at baseline (SOC: 717,000 (463,038–876225) pg/ml vs. 626,250 (520,575–946912) pg/ml, p = 0.892, Fig. 3A). However, TFPI concentrations were again lower in the TPE than in the SOC group 6 h after study inclusion (SOC: 787,175 (498,787–969,863) pg/ml vs. 500,550 (406,425–671663) pg/ml, p = 0.022, Fig. 3A). While TFPI concentrations numerically increased in the SOC group within 6 h (p = 0.076), they decreased in the TPE group (p < 0.001) (Fig. 3A). Consequentially, relative change of TFPI was + 14.4 (−2.3 to + 30.9) % in the SOC and −20 (−32.8 to −7.9) % in the TPE group (p < 0.001) (Fig. 3B), respectively. This corresponded to an absolute change in TFPI concentrations of + 60,288 (−13,369 to + 214,538) pg/ml in the SOC and −112,050 (−241,350 to −28,200) pg/ml in the TPE group (p < 0.001) (Fig. 3C), respectively.

TF/TFPI ratios were comparable between the SOC and TPE groups both at baseline (p = 0.409) and 6 h after study inclusion (p = 0.699) (Suppl. Figure 1A). Moreover, TF/TFPI ratios remained unchanged in both groups between baseline and 6 h after study inclusion (Suppl. Figure 1A). Consequentially, neither relative change (p = 0.471) (Suppl. Figure 1B) nor absolute change (p = 0.992) (Suppl. Figure 1C) in TF/TFPI ratios over time were different between SOC and TPE groups.

### Prediction of lactate response to TPE stratified by TF and TFPI elevation

**Fig. 4:**
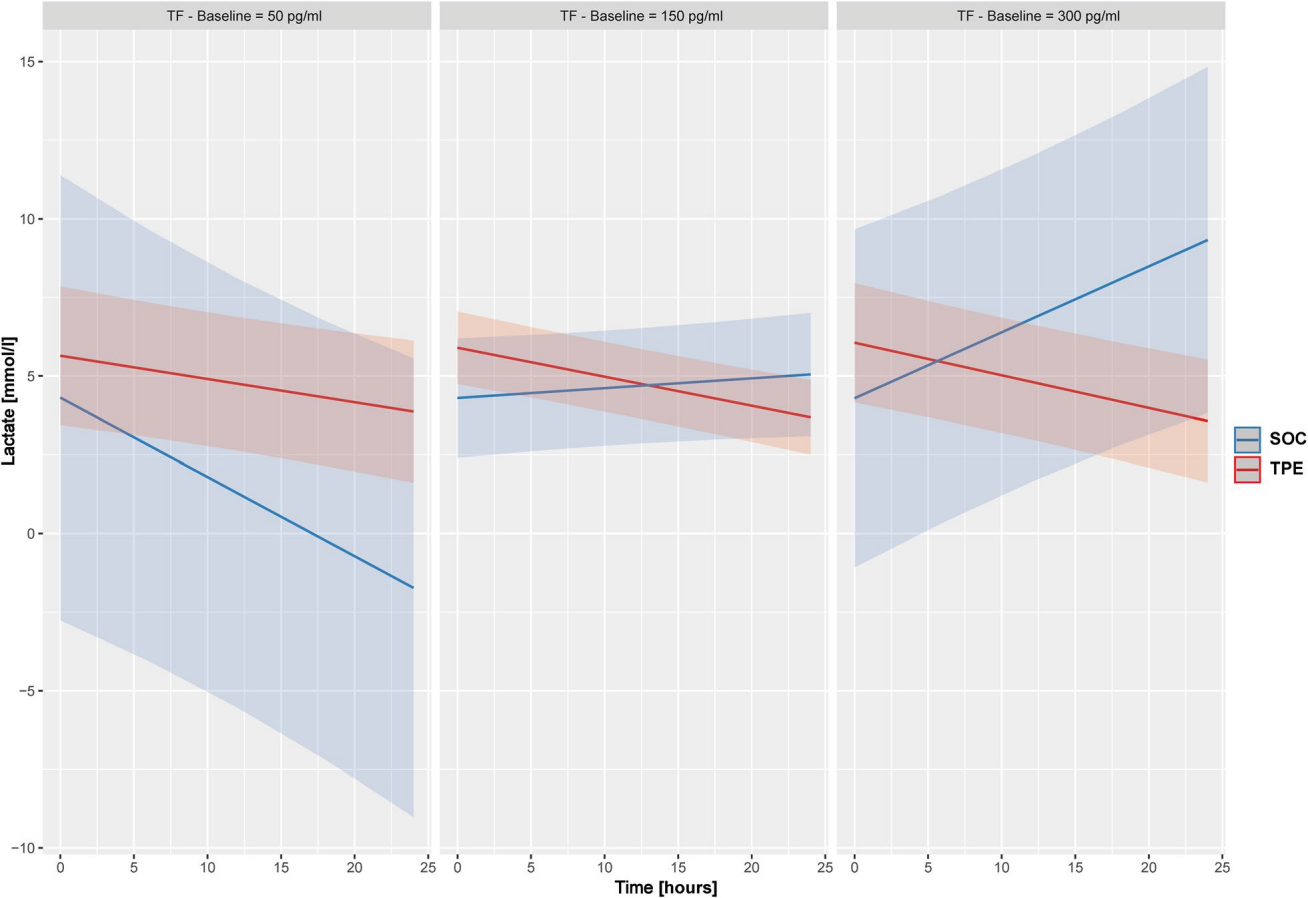
Prediction of longitudinal lactate concentration response to therapeutic plasma exchange stratified by baseline tissue factor elevation*.* Shown are estimated longitudinal lactate concentrations for both the standard of care (SOC) and therapeutic plasma exchange (TPE) group stratified by different baseline tissue factor (TF) concentrations at study inclusion. Estimated values were calculated using a triple interaction model with TPE/SOC and time, as well as all simple interactions terms between fixed effects. The model indicates that SOC patients with increasing TF concentrations experienced diminishing lactate concentration reductions over 24 h in contrast to patients under TPE which experienced sustained lactate reductions across all levels of TF (p = 0.003). At baseline TF concentrations of 50 pg/ml both groups exhibited a reduction in lactate concentrations (left panel). Above TF concentrations of 150 pg/ml, patients in the SOC group showed no lactate reduction, whereas NE reduction was maintained in the TPE group (middle panel). Above 300 pg/ml, patients in the SOC group showed increasing lactate concentrations over time, while lactate reduction was further maintained in the TPE group (right panel). The thresholds for baseline TF concentrations employed were chosen post hoc in order to best illustrate the continuous effect of TF concentrations within the model

We observed that the natural course of microcirculatory dysfunction expressed by the course of lactate levels was dependent on initial TF level. If baseline TF was relatively low (Fig. 4, left two columns, blue line), longitudinal lactate levels were stable or even decreased under SOC. To the contrary, if baseline TF was high (Fig. 4, right column, blue line), lactate levels further increased over the next 24 h under SOC. Patients undergoing TPE experienced a sustained longitudinal reduction of lactate concentrations across all levels of baseline TF elevations (Fig. 4, red line). Specifically, with a baseline TF concentration of 150 pg/ml, the slopes of lactate reduction between TPE and SOC became distinct. Above TF levels of 300 pg/ml, patients in the SOC group showed an increase in lactate concentrations, while lactate reduction was maintained in the TPE group (Fig. 4). A linear mixed effects model showed a significant triple interaction of time, study intervention and baseline TF concentration on longitudinal lactate concentrations over the first 24 h following study inclusion (p = 0.003, Suppl. Table 1).

**Fig. 5:**
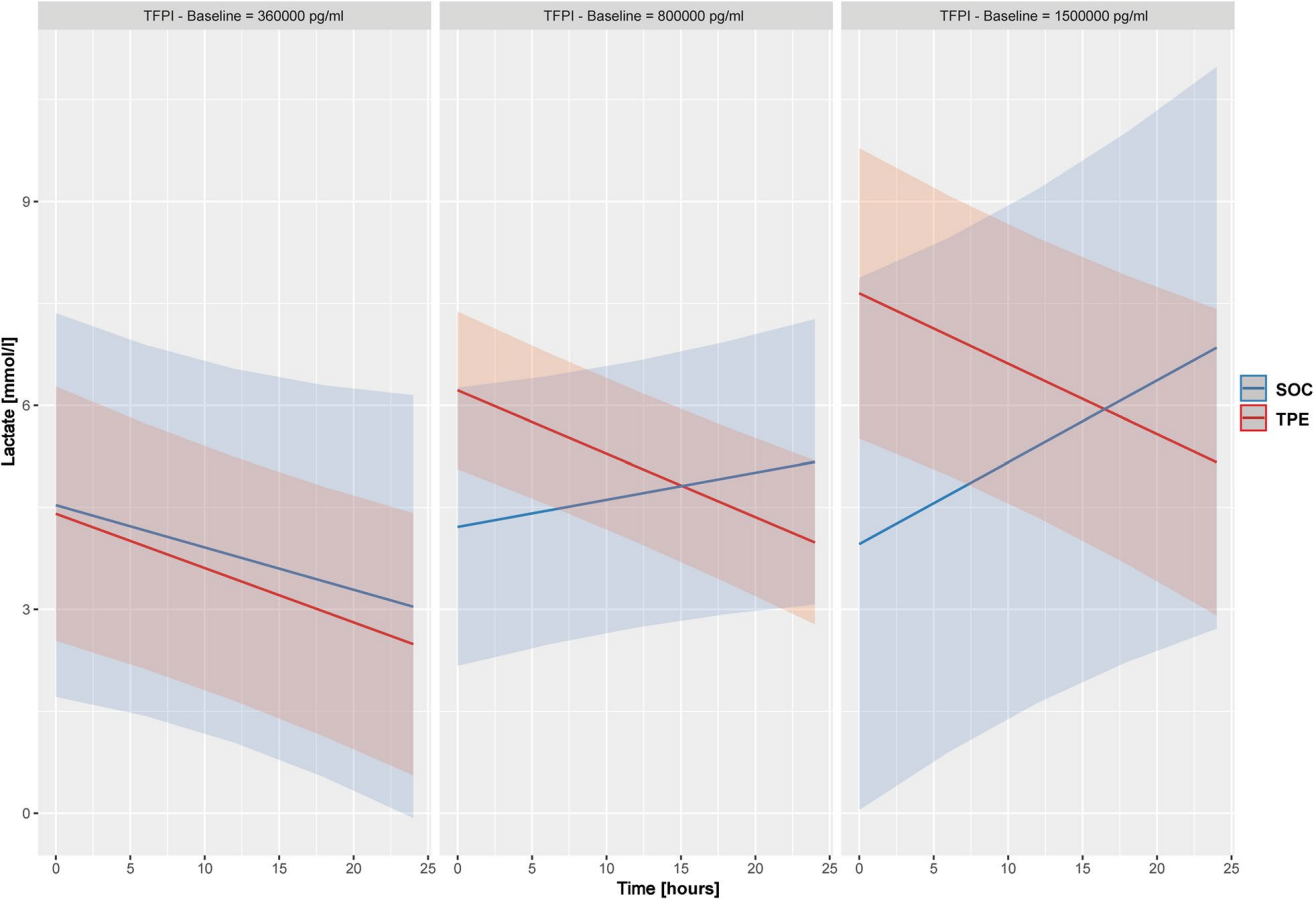
Prediction of longitudinal lactate concentration response to therapeutic plasma exchange stratified by baseline tissue factor pathway inhibitor elevation*.* Shown are estimated longitudinal lactate concentrations for both the standard of care (SOC) and therapeutic plasma exchange (TPE) group stratified by different baseline tissue factor pathway inhibitor (TFPI) concentrations at study inclusion. Estimated values were calculated using a triple interaction model with TPE/SOC and time, as well as all simple interactions terms between fixed effects. The model indicates that SOC patients with increasing TF concentrations experienced diminishing lactate concentration reductions over 24 h in contrast to patients under TPE which experienced sustained lactate reductions across all levels of TFPI (p = 0.053). At baseline TFPI concentrations of 360.000 pg/ml both groups exhibited a reduction in lactate concentrations (left panel). Above TFPI concentrations of 800.000 pg/ml, patients in the SOC group showed no lactate reduction, whereas NE reduction was maintained in the TPE group (middle panel). Above 1.500.000 pg/ml, patients in the SOC group showed increasing lactate concentrations over time, while lactate reduction was further maintained in the TPE group (right panel). The thresholds for baseline TFPI concentrations employed were chosen post hoc in order to best illustrate the continuous effect of TFPI concentrations within the model

A comparable effect on longitudinal lactate reduction was seen when response was stratified by baseline TFPI concentration (Fig. 5). Patients in the SOC group exhibited an increase in lactate concentrations over the initial 24 h when TFPI concentrations were higher at baseline (Fig. 5). In contrast, patients in the TPE group showed a sustained longitudinal reduction of lactate concentrations irrespective of baseline TFPI (Fig. 5). With a baseline TFPI concentration of 800,000 pg/ml, the slopes of lactate reduction between TPE and SOC became distinct. Above TFPI levels of 1,500,000 pg/ml, patients in the SOC group showed an increase in lactate concentrations, whereas lactate reduction was further maintained in the TPE group (Fig. 5). A linear mixed effects model showed a borderline significant triple interaction of time, study intervention and baseline TFPI concentration on longitudinal lactate concentrations over the first 24 h following study inclusion (p = 0.053, Suppl. Table 2).

## Discussion

This pooled post-hoc analysis of a prospective observational trial and a bicentric RCT examined the effects of TPE on TF and TFPI in septic shock patients. In contrast to SOC, we found that TPE reduced the levels of both TF and TFPI without affecting the TF to TFPI ratio. In a multivariate mixed-effects model, higher baseline TF (and to lesser extent TFPI) concentrations predicted early treatment response indicated by sustained longitudinal lactate reduction in the TPE group.

Higher plasma concentrations of both TF (6–9)—as well as TFPI (12) have been correlated with occurrence of DIC, multi-organ dysfunction and increased mortality in patients with sepsis. This underlines that the removal of these biologically active biomarkers and therefore concurrently disease mediators could be of therapeutic interest. Of note, the ratio of TF to TFPI was unchanged by the intervention, e.g. was not reduced by TPE as both TF and TFPI concentrations were reduced at the same time. This might be of additional significance as a recent analysis suggested especially lower TF to TFPI ratios to be closely correlated to inferior survival in septic shock patients (12).

Due to the heterogeneity of the sepsis syndrome and the study populations investigated, precision medicine approaches, including prognostic and predictive enrichment, have more recently been applied to further subgroup patients based on their individual disease biology (31–33). Predictive enrichment aims at validating a subgroup that will benefit most from a specific therapy by identifying treatable traits (34). We have previously reported that a continuous effect of TPE on the reduction of longitudinal NE doses and baseline lactate levels was predictive for such a treatment response (NE reduction) (7). In this study, we now further investigated prediction of early treatment response to TPE indicated by longitudinal lactate reduction as a surrogate of improved microcirculatory flow—an endpoint that we found relevant in regard to the target, i.e. coagulopathy. In a triple interaction term with TPE/SOC and time, baseline TF (and to a lesser extent also TFPI) plasma concentration before initiation of treatment emerged as a significant predictor of lactate response to TPE. Higher baseline TF (and TFPI) levels correlated with sustained lactate reduction in the TPE but not in the SOC group within the first 24 h following study inclusion.

This association between high TF levels and the effectiveness of TPE in reducing lactate has a plausible pathophysiologic explanation. TF is a potent initiator of the coagulation system and elevated levels seem to contribute significantly to coagulopathy and microvascular alterations in sepsis (25–29). Therefore, reduction of systemically circulating TF through TPE might mitigate this procoagulatory stimulus, especially in cases with inadequately high TF levels, thereby improving microcirculation as evidenced by the sustained lactate reduction. Our finding that the effectiveness of TPE is related to high TF levels is highly relevant, given that all other treatment approaches aimed at modulating coagulation in sepsis (30–32), such as those targeting the TF-TFPI pathway (11), have failed to show a consistent benefit in sepsis patients, despite being more specific then TPE.

This study has limitations; first its relatively small sample size and bicentric setting affecting the power of the results. A further concern is the quite complex post-hoc study design with a numerical imbalance between the two groups (with and without TPE) since patients` biomaterials had to be pooled from both a non-randomized observational study and an open label RCT. Together with the fact that biomaterial was missing for the present analysis from nine of the original 60 patients treated in both studies, this results in comparison of only 14 SOC to 37 TPE patients, therefore certainly introducing a potential selection bias. However, the patient cohorts of the EXCHANGE-Pilot and EXCHANGE-1 studies were quite comparable in terms of both demographic characteristics and clinical disease severity at baseline due to the same inclusion criteria used in both consecutively performed studies (13–15). Furthermore, the here analyzed subset of patients receiving SOC was still comparable to patients receiving additional TPE in all demographic and most clinical disease severity parameters (e.g. SOFA Score, Lactate concentration, extent of RRT and mechanical ventilation, oxygenation index). Of note, the NE dose at baseline was higher in the TPE than the SOC group, potentially introducing a further bias.

The intervention was administered in a singular session and at a fixed dose, which precludes us from providing data on effects at different dosages or time frames. Since TPE was performed within 6 h of randomization, TPE could have been performed very early (first hour) or late (hours 5 to 6) within this timeframe potentially influencing biomarker levels and treatment efficacy. Repeated measurements at preselected timepoints including the days following treatment should be implemented in follow-up trials to capture the variability due to biomarker assessment time (33). Last but not least, this study only applies to severe refractory septic shock with exceedingly high doses of norepinephrine, therefore only representing a smaller subset of septic shock patients. Finally, both studies have been performed from 2016 to 2020, therefore before the current surviving sepsis guidelines of 2021 (3), potentially influencing standard of care treatment applied in these studies.

In summary, the current study, despite its post-hoc pooled design potentially introducing selection bias, suggests that adjunctive TPE therapy in septic shock is associated with a significant removal of both TF and TFPI, which may contribute to the early hemodynamic improvement observed in septic shock patients. Higher baseline TF (and TFPI) plasma concentrations were identified as putative predictors of early treatment response of microcirculation, i.e. longitudinal lactate reduction, that could be useful for predictive enrichment strategies in future clinical trials.

## Supplementary Information

Supplementary material 1

Supplementary material 2

Supplementary material 3

## Data Availability

The datasets used and analyzed are during the current study are available from the corresponding author on reasonable request.

## Abbreviations

AKI: Acute kidney injury
BMI: Body mass index
CRP: C-reactive protein
DIC: Disseminated intravascular coagulation
FFP: Fresh frozen plasma
IQR: Interquartile range
MAP: Mean arterial pressure
NE: Norepinephrine
PCT: Procalcitonin
RCT: Randomized controlled trial
RRT: Renal replacement therapy
SIC: Sepsis-induced coagulation
SOC: Standard of care
SOFA: Sequential Organ Failure Assessment
TF: Tissue factor
TFPI: Tissue factor pathway inhibitor
TPE: Therapeutic plasma exchange
WBC: White blood cell
