# Supplementary figures and images for: Effect of therapeutic plasma exchange on tissue factor and tissue factor pathway inhibitor in septic shock
Source: Crit Care. 2024 Oct 30;28:351. doi: 10.1186/s13054-024-05142-4 (PMC11526504; doi:10.1186/s13054-024-05142-4)

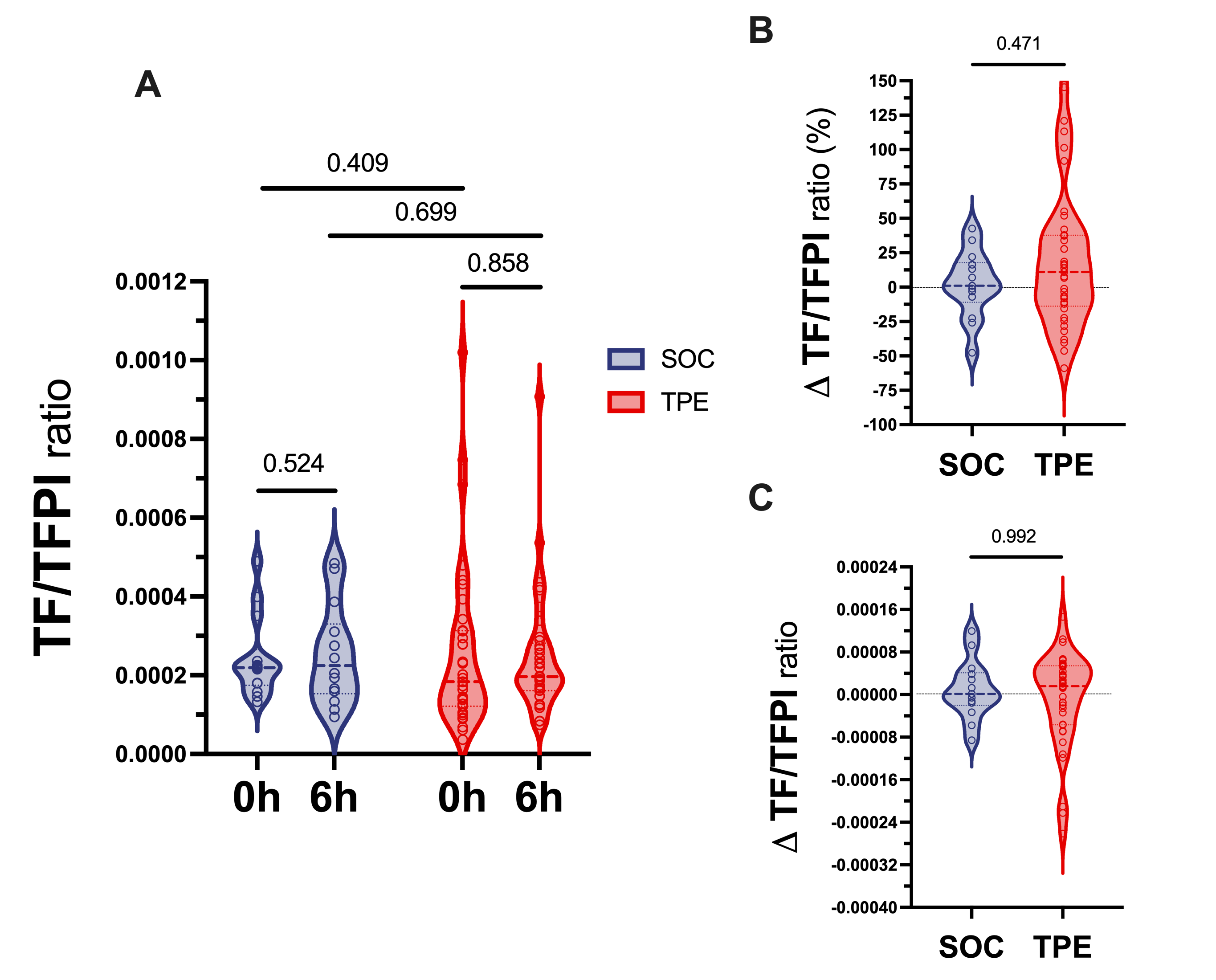

Supplement: Supplementary file 1 — Supplementary material 1 [file 13054_2024_5142_MOESM1_ESM.tiff]
