# Supplementary material for: Effect of therapeutic plasma exchange on tissue factor and tissue factor pathway inhibitor in septic shock
Source: Crit Care. 2024 Oct 30;28:351. doi: 10.1186/s13054-024-05142-4 (PMC11526504; doi:10.1186/s13054-024-05142-4)
Supplement: Supplementary file 2 — Supplementary material 2 [file 13054_2024_5142_MOESM2_ESM.docx]

| **Suppl. Table 1**: Linear mixed-effect model for the prediction of longitudinal lactate concentrations stratified by baseline tissue factor concentration | | | |
| --- | --- | --- | --- |
| **Predictors** | **Estimates** | **99% confidence interval** | **p** |
| Intercept | 4.35 | -28.44 – 37.15 | 0.794 |
| TPE | 0.38 | -33.74 – 34.5 | 0.982 |
| TF baseline [log] | -0.01 | -6.64 – 6.61 | 0.997 |
| Time | -1.26 | -2.13 – 0.39 | **0.005** |
| Interaction: TPE x TF baseline [log] | 0.24 | -6.65 – 7.14 | 0.945 |
| Interaction: TPE x Time | 1.25 | 0.35 – 2.15 | **0.007** |
| Interaction: TF baseline [log] x Time | 0.26 | 0.08 – 0.43 | **0.004** |
| *Interaction: TPE x TF baseline [log] x Time* | *-0.27* | *-0.46 – -0.09* | ***0.003*** |
