# Supplementary material for: Effect of therapeutic plasma exchange on tissue factor and tissue factor pathway inhibitor in septic shock
Source: Crit Care. 2024 Oct 30;28:351. doi: 10.1186/s13054-024-05142-4 (PMC11526504; doi:10.1186/s13054-024-05142-4)
Supplement: Supplementary file 3 — Supplementary material 3 [file 13054_2024_5142_MOESM3_ESM.docx]

| **Suppl. Table 2**: Linear mixed-effect model for the prediction of longitudinal lactate concentrations stratified by baseline tissue factor pathway inhibitor concentration | | | |
| --- | --- | --- | --- |
| **Predictors** | **Estimates** | **99% confidence interval** | **p** |
| Intercept | 9.68 | -43.27 – 62.62 | 0.719 |
| TPE | -34.31 | -95.9 – 27.27 | 0.273 |
| TFPI baseline [log] | -0.4 | -4.37 – 3.56 | 0.842 |
| Time | -1.7 | -3.42 – 0.02 | 0.053 |
| Interaction: TPE x TFPI baseline [log] | 2.67 | -1.93 – 7.28 | 0.254 |
| Interaction: TPE x Time | 1.83 | 0.13 – 3.79 | 0.067 |
| Interaction: TFPI baseline [log] x Time | 0.13 | 0.00 – 0.26 | 0.051 |
| *Interaction: TPE x TFPI baseline [log] x Time* | *-0.14* | *-0.29 – -0.00* | *0.053* |
